# Don’t Turn Blind! The Relationship Between Exploration Before Ball Possession and On-Ball Performance in Association Football

**DOI:** 10.3389/fpsyg.2018.02520

**Published:** 2018-12-10

**Authors:** Thomas B. McGuckian, Michael H. Cole, Geir Jordet, Daniel Chalkley, Gert-Jan Pepping

**Affiliations:** ^1^School of Behavioural and Health Sciences, Australian Catholic University, Brisbane, QLD, Australia; ^2^Department of Coaching and Psychology, Norwegian School of Sport Sciences, Oslo, Norway

**Keywords:** soccer, performance analysis, affordance, decision making, vision, ecological psychology, spatial awareness, scanning

## Abstract

Visual exploratory action – scanning movements expressed through left and right rotation of the head – allows perception of a surrounding environment and supports prospective actions. In the dynamically changing football environment, the extent to which exploratory action benefits a player’s subsequent performance with the ball is likely influenced by how and when the exploratory action occurs. Although few studies have examined the relationship between visual exploration and on-pitch football performance, it has been reported that a higher frequency of exploratory head movement up to 10-s before receiving the ball increases the likelihood of successful performance with the ball. This study investigated the relationship between head turn frequency and head turn excursion, and how and when exploratory head movement – within 10-s before ball possession – is related to performance with the ball in 11v11 match-play. Thirty-two semi-elite football players competed in 11v11 match-play. Head turn frequency and head turn excursion before ball possession were quantified with wearable inertial measurement units, and actions with the ball were coded via notational analysis. Odds ratio calculations were conducted to determine the associations between exploration variables and on-ball performance outcomes. A total of 783 actions with the ball were analyzed. Results revealed a strong relationship between head turn frequency and head turn excursion. Further, a higher than average head turn frequency and head turn excursion before receiving the ball resulted in a higher likelihood of turning with the ball, playing a pass in the attacking direction, and playing a pass to an area that is opposite to which it was received from. The strength of these outcomes varied for different time periods before receiving the ball. When players explored their environment with higher than average head turn frequency and excursion, they used more complex action opportunities afforded by the surrounding environment. Considerations for future research and practical implications are discussed.

## Introduction

“Don’t turn blind” or “check your shoulder” is often exclaimed by coaches when a player unwittingly turns into an opponent, usually resulting in the loss of possession. Given the dynamic nature of football match-play (Memmert et al., 2017), performance is determined by a complex interaction between various physical, psychological, technical, and tactical components (Hughes et al., 2012; Lovell et al., 2018; Sarmento et al., 2017; Brink et al., 2018). Therefore, understanding specific determinants of performatory actions with the ball can be difficult, as the events that lead to a successful pass, for example, will be a complex combination of the physical, psychological, technical, and tactical components of the game for that specific pass. Performance analysis in football has typically focussed on player actions with the ball (Carling et al., 2005; Sarmento et al., 2014), as variables such as possession (Castellano et al., 2012; Liu et al., 2015), pass accuracy (Redwood-Brown, 2008; Rampinini et al., 2009; Liu et al., 2016), shots on goal (Rampinini et al., 2009; Castellano et al., 2012; Liu et al., 2016), and pass effectiveness (Rein et al., 2017) have been related to overall performance. Given that actions with the ball are so valuable for overall match success, it is important to also understand the factors that lead up to them. Whilst coaches and players know intuitively that it is important to visually scan their surroundings in order to aid performance (Pulling et al., 2018), to date, little research has been devoted to understanding how players visually perceive their surroundings, and how this relates to on-pitch football performance.

In order for a player to perform a successful action with the ball in football, they must prospectively guide their actions (Reed, 1996; Adolph et al., 2000; Fajen, 2007; Fajen et al., 2009). To do this, players engage in visual exploratory actions, in which they move their body, head, and eyes to visually perceive the game around them (Gibson, 1966, 1979; Reed, 1996). In doing so, the player is able to perceive the availability of space and other players, which provides information about the opportunities to act (known as affordances; Gibson, 1979; Fajen et al., 2009), such as an open pass, space to run into, or time to shoot. Critically, players can engage in this exploratory action *before* they receive the ball, which allows the guidance of their performatory actions with the ball once they have gained possession (McGuckian et al., 2018b). This visual exploratory action, therefore, is an important consideration when analyzing performatory actions with the ball in football.

Previous investigations have shown that an increase in a player’s exploratory action prior to receiving the ball, expressed as a higher frequency of head movements, resulted in improved performance with the ball. These improvements in performatory actions include faster subsequent passes (McGuckian et al., 2018b), more turns with the ball and more forward passes (Eldridge et al., 2013), and a higher likelihood of playing a successful pass (Jordet et al., 2013). Taken together, this research shows the importance of visually scanning one’s surroundings *prior* to receiving the ball. Despite these encouraging findings, the exploratory head movements of football players have been scarcely investigated during live match-play (McGuckian et al., 2018c), indicating a need to strengthen the evidence base with investigations that comprehensively capture the exploratory actions used by players in competitive settings.

In football, the constant movement of two teams of 11 players results in a dynamic and complex environment in which players compete (Memmert et al., 2017). As a consequence, a pass to a teammate that is afforded in one instance may not be afforded to the player in the next instance (Fajen, 2007; Fajen et al., 2009). Given their constantly changing environment, it is important to understand when, in the time that players spend before ball possession, visual exploration is most important for performance with the ball. Jordet et al. (2013) investigated the visual exploratory head movements that occurred in the 10-s before ball possession and found that a higher frequency of head movements increased the chance of a successful pass to a teammate (i.e., successful performatory action). However, affordances for a footballer can change drastically in 10-s, and it may be that exploratory action in a shorter time period before ball possession could be more important for subsequent successful performance with the ball. McGuckian et al. (2018b) investigated exploratory actions 1, 2, and 3 s before ball possession, and showed that players were able to respond with a pass more quickly when they had longer (2 or 3 s) to explore before gaining ball possession; that is, a lack of time to explore (i.e., 1 s of exploration) did not allow enough time for the players to adequately determine future opportunities for action. With a better understanding of the optimal time-periods to explore before a player gains possession of the ball, practitioners can better focus their assessment and development of exploratory head movement in applied settings.

To allow an extended understanding of the behavior in the information rich environment that players compete, previous definitions of visual exploratory action in football require further elaboration. Previously, visual exploration in a football context has been operationally defined as movements of the “body and/or head prior to receiving the ball, engaged in to perceive information away from the ball and to act appropriately when the ball arrives” (Jordet, 2005, p. 141). While logical, this definition is somewhat subjective and may inadvertently miss important qualitative information about the exploratory head movements used by footballers. For example, a player who looks away from the ball to their right, then further to their right before looking back toward the ball, may be picking up different environmental information (and therefore affordances) than when that same player only looks away from the ball to their right and straight back to the ball. The first situation could be described as a *sequential exploration* (Jordet, 2005), and it is functionally and kinematically distinct to the second situation. Consequently, there is a need to supplement the operational definition used previously by defining exploratory action by the movement itself. McGuckian et al. (2018b) defined visual exploratory action as “a distinct movement of the head about the longitudinal axis” (p. 8). Similarly, Chalkley et al. (2018) validated the measurement of exploration as head movement that occurs around the longitudinal (yaw) axis. Definition of visual exploration for information in situations where individuals are surrounded by information in this manner provides a relevant and complete representation of the exploratory head movements used by players (in the yaw orientation), and it covers both sequential and non-sequential head movements. Therefore, the definition given by McGuckian et al. (2018b) and validated by Chalkley et al. (2018) has been adopted here.

Another important aspect of visual exploration in football, related to *how much* of the environment a player has explored, is the total radial distance of a head turn, here termed *excursion*. Despite giving an understanding of the magnitude of a head turn, and being recognized to be an important aspect of exploration (Jordet and Pepping, 2018; McGuckian et al., 2018a,b), previous quantification of exploration through manually counting head movements is unable to accurately quantify excursion. For this reason, exploration excursion has thus far not received any attention in the scientific literature.

Building upon previous research, inertial measurement units (IMUs) have recently been validated to quantify exploratory head movements (Chalkley et al., 2018), and have been used in representative laboratory environments (McGuckian et al., 2018a,b). Housed in an elastic headband and worn at the back of the player’s head, these devices allow time-efficient data collection and analysis on whole teams of players and provide sensitive and objective data collection that is able to detect the rapid head movements used by footballers in live match-play. Together, the variables obtained from head-mounted IMUs reported in this study give an indication of *how often* a player explores their environment (i.e., the rate of exploration – frequency) and *how much* of the environment a player explores (excursion) before gaining possession of the ball (McGuckian et al., 2018a).

**FIGURE 1:**
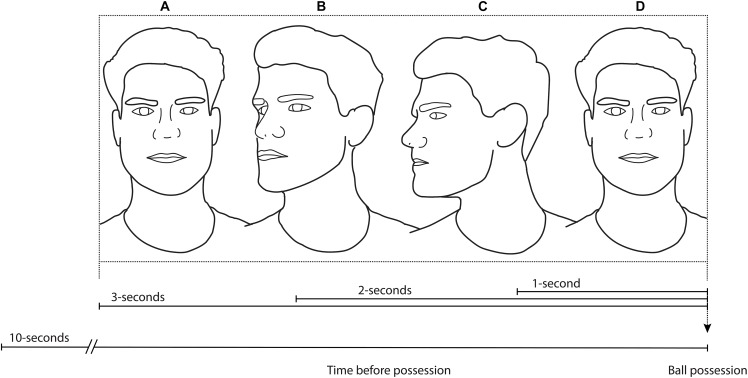
An example of three head turns during a 3-s period of time before possession: One head turn from **(A,B)** (approx. 45 degrees), one head turn from **(B,C)** (approx. 45 degrees), and one head turn from **(C,D)** (approx. 90 degrees). For this 3-s period, the head turn frequency is equal to 1.0 (three turns/three seconds) and the head turn excursion is equal to 60-degrees per second (180-degrees/three seconds).

Despite the recent interest in exploratory head movement of footballers, it is unclear how and when – in such a fast-paced and constantly changing environment – exploratory head movements before ball possession are related to performance with the ball in 11v11 match-play. To prepare for elaborate research questions that include football specific individual differences and contexts, a general understanding of the relationships between exploration and on-ball performance is required. To that end, the primary aim of this study was to further our understanding of how exploratory head movements relate to performance with the ball, and how this relationship changes with exploration in various time-periods before gaining possession of the ball. In doing so, the frequency and excursion of football players’ head movements before ball possession were quantified, in 10 increasing time-periods, up to 10-s before ball possession (i.e., 0–1 s before ball possession; 0–2 s before ball possession, etc., up to 0–10 s before ball possession; see also Figure 1). Following previous research (Eldridge et al., 2013; Jordet et al., 2013; Pocock et al., 2017), exploratory head movements were then related to common important measures of on-ball performance. It was expected that increased head turn frequency and head turn excursion before gaining possession of the ball would result in a higher likelihood of successful actions with the ball. It was further expected that exploratory head movement performed closer to the time of ball reception would be more closely associated with on-ball performance than temporally distant exploration.

This study is the first of its kind to consider the frequency and excursion of exploratory head movements. Hence, the relationship between the frequency and excursion of head movements have not previously been examined. Therefore, a secondary aim of this study was to investigate the relationships between these two exploratory variables in the defined time-periods before ball possession. It was expected that head turn frequency and head turn excursion would be closely related across all time-periods before ball possession. That is, it was hypothesized that as the frequency of head turns increases the total excursion of head turns also increases.

## Materials and Methods

### Participants

Participants were 32 male football players aged 16–30 years (19.03 ± 2.88 years). The sample represented all regular outfield playing positions. Goalkeepers were not included in the study due to the specificity of their role. Participants were conveniently recruited from clubs playing in the Australian National Premier League, therefore representing a group with homogeneous playing ability. The participants were assessed to be at the standard of semi-elite players in Australia (Swann et al., 2015). To be included in the study, participants needed to be considered free from injury by club medical staff. In accordance with the Declaration of Helsinki, written informed consent was obtained from all adult participants and the parents/legal guardians of all non-adult participants. The protocol was approved by the Australian Catholic University Human Research Ethics Committee (Application ID: 2017-154H) and participants were free to withdraw at any stage.

### Procedure

Participants competed in 11v11 match-play according to official rules (International Football Association Board, 2017), with some minor adaptations. Given that players wore an IMU on their head, matches could not be officially sanctioned by the governing body. Instead of collecting data in competitive matches, participants played in pre-season games in which final team selection was not yet decided. Additionally, as a pre-season load management precaution, matches were not all played as two halves of 45-min each. Instead, play was divided into two or three playing periods that ranged from 20 to 45 min each, resulting in total match times of between 60 and 80 min. Despite the above adaptations, the games were deemed to be acceptably competitive and representative of competitive match-play.

Outfield players wore a 9-degrees-of-freedom IMU (SABELSense, Nathan, QLD, Australia), housed within an elastic headband, over their occipital protuberance while playing. Data were captured at 250 Hz and stored locally on each IMU’s memory card. Following the completion of games, data were downloaded and processed using a custom-made algorithm developed in MATLAB (MathWorks, Natick, MA, United States). This previously validated algorithm determines the time at which a distinct head turn occurs, defined as a movement of the head about the longitudinal axis that exceeds 125 deg/s (Chalkley et al., 2018; McGuckian et al., 2018b). Further, the algorithm determines the total excursion (i.e., angular distance) of each head turn by finding the absolute difference in orientation of the IMU between the beginning and end of each head turn. Together, the outputs from the head-mounted IMU gave an indication of how often a player explored the field and how much of the field the player explored.

Matches were video recorded with two high-definition video cameras (Sony FDR-AX100E, Tokyo, Japan) at 50 Hz from an elevated position along the side of the playing area. One video camera was zoomed close to the ball, while the other was zoomed out to give a wide angle of the playing field. The combination of the two camera angles ensured quality footage to assist with notational analysis of performance with the ball.

### Variables

The two video sources were synchronized with each player’s IMU data to allow manual coding of actions with the ball and the calculation of variables used for statistical analysis.

#### Exploratory Action

##### Head turn frequency (HTF)

The total number of head turns – as obtained from the IMU data – were divided by the number of seconds in that time-period, giving the frequency of head turns before ball possession for each time-period before ball possession (see Figure 1).

##### Head turn excursion (HTE)

The excursion of each head turn – as obtained from the IMU data – was summed to give a total excursion for each time-period before ball possession. In order to time-normalize excursion and therefore compare different time-periods, the total HTE was then divided by the number of seconds in the time-period, therefore expressing HTE in total degrees per second of play (see Figure 1).

#### Performance With the Ball

Each players’ possessions – as coded in SportsCode v.11.2.15 (Hudl, Lincoln, NE, United States) – were tagged with technical on-ball performance indicators commonly used in performance analysis research (Hughes and Franks, 2005; Dellal et al., 2011; Russell et al., 2013; Morgans et al., 2014; Liu et al., 2016). An operational definition, including the total number of occurrences across all participants, of each of the performance variables are given in Table 1. Together, these tagged variables described the players’ actions with the ball and were treated as outcome variables in the statistical analysis.

**Table 1:** Operational definition of each performance outcome.

Performance Outcome	Coded action	Definition	Total number of occurrences
*Pass direction – Field-centric (PD-FC)*	Pass forward	The direction of a pass is in the team’s attacking direction	265
	Pass backward	The direction of a pass is in the team’s defensive direction	106
*Pass direction – Player-centric (PD-PC)*	Pass behind	The direction of a pass is behind the player relative to the area it was received. For example, when a player receives a pass from one side of the field and plays a pass to the other side of the field	172
	Pass return	The direction of a pass is returned back toward the area to which it was received. For example, when a player receives a pass from one side of the field and plays a pass back to the same side of the field	141
*Turn with the ball*	Turn with the ball	After receiving the ball, the player turns with the ball in order to move in a different direction	261
	No turn with the ball	After receiving the ball, the player completes a subsequent action without turning with the ball	522
*One-touch pass*	One-touch pass	A pass in which the player requires no more than one touch (i.e., the pass itself) to deliver the ball to a teammate. Only possessions in which a pass was attempted were included	168
	Not one-touch pass	A pass in which the player takes at least one touch to control the ball and another to pass the ball (i.e., at least two touches). Only possessions in which a pass was attempted were included	467
*Pass success*	Successful pass	An intentionally played ball which successfully reaches a teammate and possession is retained	549
	Unsuccessful pass	An intentionally played ball which goes out of bounds or is intercepted by the opposition and results in losing possession	71

*Pass direction – Player-centric can include passes in all field-centric directions (i.e., a pass behind and a pass return can also be a pass forward or a pass backward)*.

#### Time Before Possession

The exploratory actions of each player were collected for the time prior to each individual ball possession for ten time-periods, spanning from 1-s before ball possession to 10-s before ball possession (see Figure 1). The players were deemed to be in possession of the ball once their foot (or other legal body part) first made contact with the ball. For a one-touch pass, the time at which foot-to-ball contact was made was also used to signify the moment of ball possession.

### Statistical Analysis

#### Inter-Rater Reliability

To ensure accurate coding of performance with the ball, 159 of the 783 possessions (20%) were coded by a second coder. Inter-rater reliability (IRR) analysis was performed to determine the degree of consistency between the two coders’ assessments of performance. Kappa values (Cohen, 1960) were calculated for each performance measure presented in the results. For pass direction – player centric (PD-PC) and pass direction – field centric (PD-FC), kappa values were calculated for each pass direction and averaged to provide a single IRR value (Landis and Koch, 1977). Kappa values indicated substantial agreement between coders for PD-PC [*k* = 0.724, 95% CI (0.598, 0.850)] and turns with the ball [*k* = 0.641, 95% CI (0.522, 0.760)], and almost perfect agreement for pass success [*k* = 0.835, 95% CI (0.758, 0.912)], PD-FC [*k* = 0.854, 95% CI (0.767, 0.940)] and one-touch pass [*k* = 0.817, 95% CI (0.720, 0.914)].

#### Descriptive Analysis

One-way repeated measures analyses of variance (ANOVA) were conducted to compare the effect of time before possession (ten levels) on the average HTF and HTE. When Mauchly’s test indicated the assumption of sphericity had been violated, the Greenhouse-Geisser correction was used to adjust the degrees of freedom. Post-hoc comparisons were completed on adjacent levels using Bonferroni tests. To investigate the relationship between HTF and HTE, exploratory Pearson’s correlation tests were run on the HTF and HTE for each time-period before possession. Alpha was set at *p* < 0.05.

#### Relationship Between Exploration and Performance With the Ball

Research has demonstrated that individual players show differences in the frequency of head turns before ball possession (McGuckian et al., 2018a). Therefore, we normalized data to input into odds ratio (OR) calculations by comparing each player’s HTF and HTE before each ball possession to their individual average HTF and average HTE across all of their own possessions. As a result, the HTF and HTE for each possession for each player was categorized as being either higher or lower than their individual average HTF and average HTE.

For each time period before ball possession, ORs were calculated to determine the association between exploration (higher or lower HTF and HTE) and each performance outcome as described in Table 1. The first listed action (i.e., *pass forward, pass behind, turn with the ball, one-touch pass*, and *successful pass*) for each outcome was treated as the outcome of interest. For each OR, a value above 1 indicated that the outcome of interest was more likely to occur when the players’ HTF or HTE before ball possession was higher than their individual average. In contrast, an OR below 1 indicated that the outcome of interest was more likely to occur when the players’ HTF or HTE before ball possession was less than their individual average (Schmidt and Kohlmann, 2008; Szumilas, 2010).

## Results

The mean, standard deviation and range of HTF and HTE for each time-period before ball possession, as well as the correlation between HTF and HTE, are shown in Table 2. As participants came closer to receiving the ball (i.e., time before possession approached 1-s), the mean HTF and HTE became significantly higher. There was a strong correlation between HTF and HTE for all time-periods before possession, with the strongest relationship occurring 10-s before ball possession.

**Table 2:** Mean, standard deviation and range of head turn frequency (HTF) and head turn excursion (HTE), and correlation between HTF and HTE for each time-period before possession.

Time-period before possession	Mean (SD) HTF (turns/second)	HTF range	Mean (SD) HTE (degrees/second)	HTE range	Correlation between HTF and HTE (Pearson’s *r*)
1-s	1.44 (0.53)^#^	0.50–2.96	56.11 (27.27)^#^	12.47–106.75	0.666^∗^
2-s	1.32 (0.40)^#^	0.70–2.48	50.60 (20.19)^#^	15.70–88.20	0.658^∗^
3-s	1.20 (0.34)^#^	0.67–2.04	45.64 (15.63)^#^	20.83–80.43	0.639^∗^
4-s	1.15 (0.32)^#^	0.65–1.86	43.69 (14.32)^#^	20.47–71.54	0.658^∗^
5-s	1.09 (0.28)^#^	0.59–1.71	41.43 (13.12)^#^	18.18–66.64	0.670^∗^
6-s	1.05 (0.27)^#^	0.52–1.67	39.90 (12.09)^#^	16.83–63.47	0.678^∗^
7-s	1.02 (0.25)^#^	0.51–1.53	38.63 (11.46)^#^	17.33–61.03	0.707^∗^
8-s	0.99 (0.23)^#^	0.53–1.43	37.27 (10.85)^#^	19.09–58.59	0.718^∗^
9-s	0.96 (0.22)^#^	0.50–1.36	35.96 (10.55)^#^	18.29–58.05	0.728^∗^
10-s	0.95 (0.21)^#^	0.49–1.29	35.29 (10.30)^#^	18.03–57.76	0.744^∗^

*^#^ indicates difference to adjacent time-periods at *p* < 0.05, ^∗^ indicates correlation at *p* < 0.001*.

**FIGURE 2:**
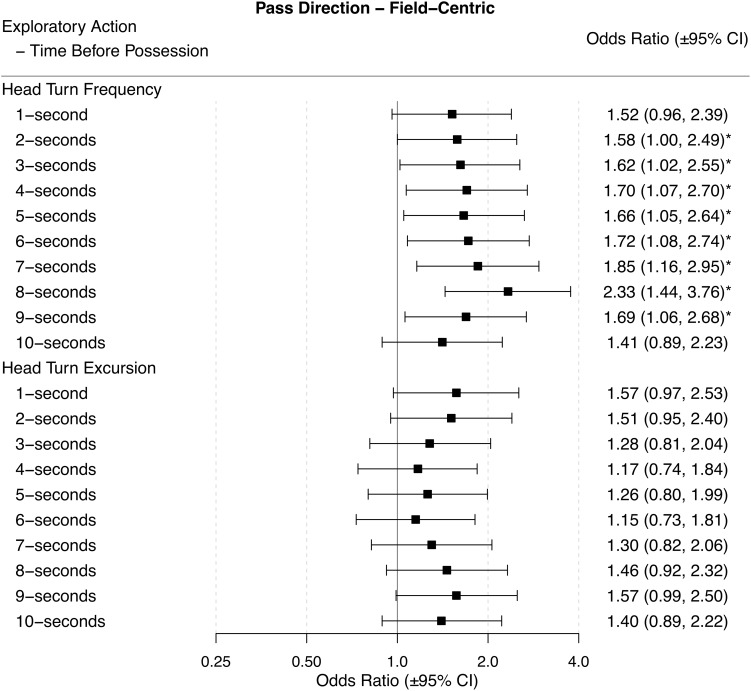
Odds ratios (±95% CI) for each time period before ball possession, describing the associations between HTF and HTE, and pass direction – field centric. ^∗^ indicates statistical significance, as the 95% CIs do not cross 1.

**FIGURE 3:**
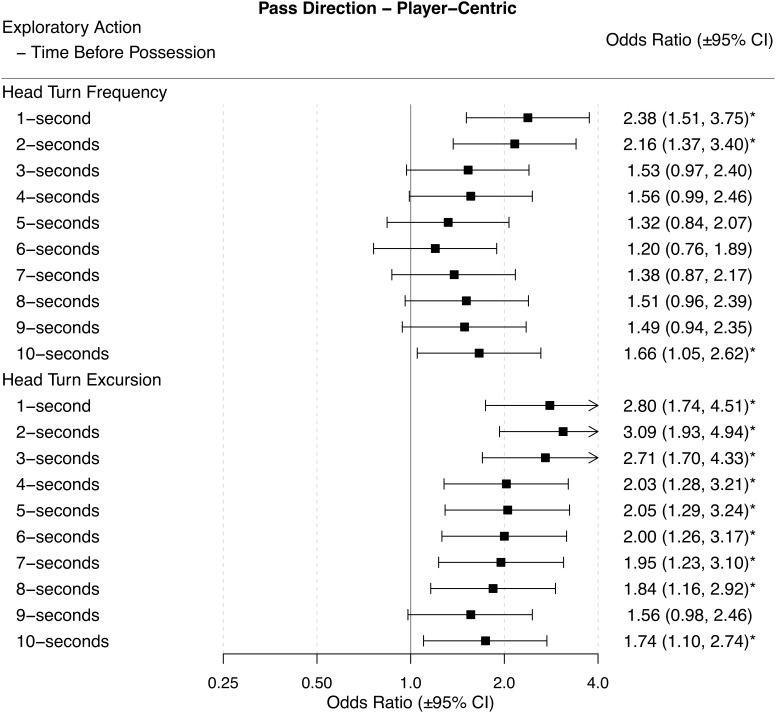
Odds ratios (± 95% CI) for each time period before ball possession, describing the associations between HTF and HTE, and pass direction – player centric. ^∗^ indicates statistical significance, as the 95% CIs do not cross 1.

**FIGURE 4:**
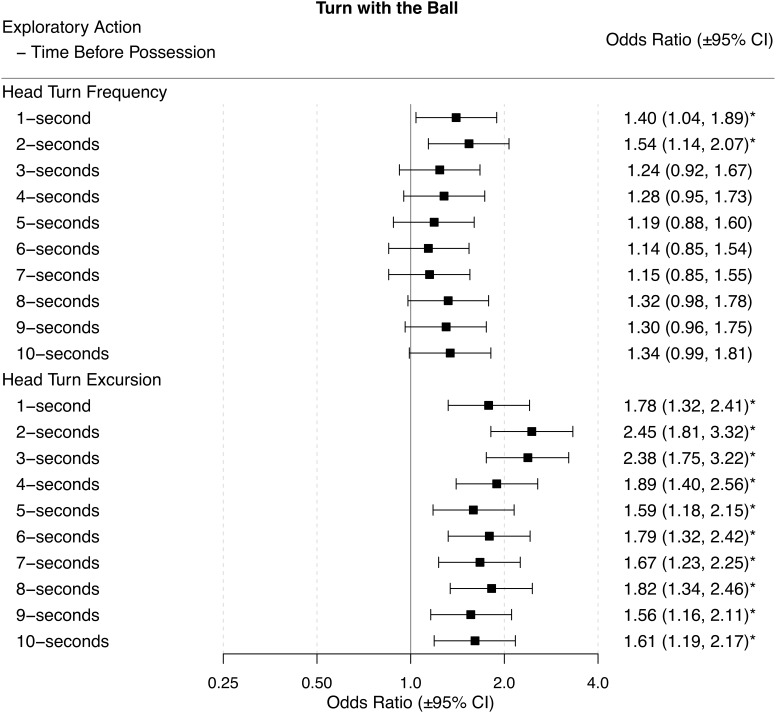
Odds ratios (±95% CI) for each time period before ball possession, describing the associations between HTF and HTE, and turns with the ball. ^∗^ indicates statistical significance, as the 95% CIs do not cross 1.

**FIGURE 5:**
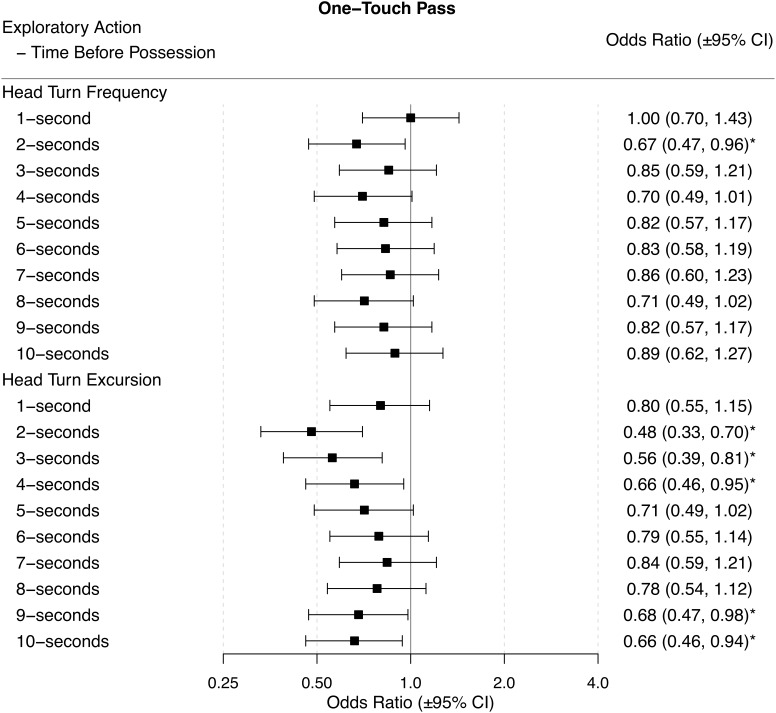
Odds ratios (±95% CI) for each time period before ball possession, describing the associations between HTF and HTE, and one-touch passes. ^∗^ indicates statistical significance, as the 95% CIs do not cross 1.

**FIGURE 6:**
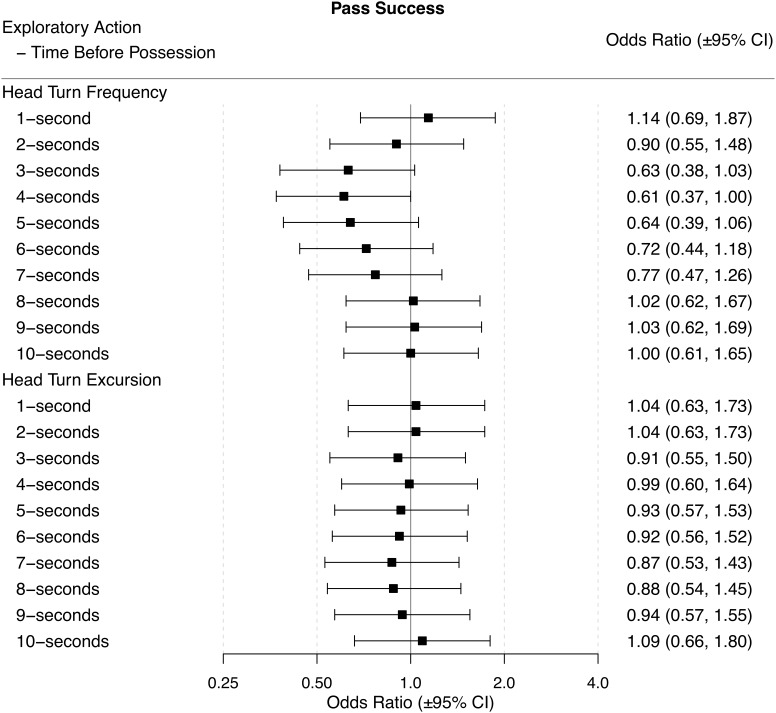
Odds ratios (±95% CI) for each time period before ball possession, describing the associations between HTF and HTE, and pass success.

Odds ratios (±95% CI) for each performance outcome across each time period before ball possession are presented in Figures 2–6.

### Pass Forward

When players had a higher HTF 2-s to 9-s before ball possession they were more likely to play a forward pass. A higher or lower HTE was not associated with a higher likelihood of playing a forward pass for any time-period before gaining possession of the ball (Figure 2).

### Pass Behind

A higher HTF 1-s, 2-s, and 10-s before gaining ball possession was associated with a higher likelihood of playing a pass to an area opposite to where it was received from (Figure 3). When players had a higher HTE before ball possession they were more likely to play a pass to an area that was opposite to where it was received from for all time-periods except for 9-s before gaining possession of the ball. Odds ratios indicated that with a higher HTE during the 1-s to 6-s before gaining ball possession, players were two to three times more likely to play a pass to an area that was opposite to where it was received from (Figure 3).

### Turn With the Ball

When players had a higher HTF in the 1-s or 2-s before gaining possession they were more likely to turn with the ball. When players had a higher HTE they were more likely to turn with the ball for all time-periods before gaining possession of the ball (Figure 4).

### One-Touch Pass

Players were more likely to play a one-touch pass when they had a lower HTF 2-s before gaining possession of the ball. Players were more likely to play a one-touch pass when they had a lower HTE in the 2-s, 3-s, 4-s, 9-s, and 10-s before receiving ball possession (Figure 5).

### Successful Pass

Neither HTF nor HTE before ball possession were associated with the likelihood of playing a successful pass for any time-period before ball possession. However, for the 3-s, 4-s, and 5-s before gaining possession of the ball, the likelihood of a successful pass with a lower HTF approached statistical significance (Figure 6).

## Discussion

With the aim of further understanding exploratory head movements before gaining ball possession and their relationship to performance with the ball, the frequency and excursion of head movements were quantified in time-periods of increasing duration, up to 10-s prior to taking possession of the ball. Given frequencies and excursions higher or lower than a player’s individual average, the likelihood of completing various actions with the ball were calculated.

Players completed approximately one head turn per second in the 5–10 s before receiving the ball, and the average excursion of these head turns was approximately 37 degrees per second. However, when players came very close to receiving the ball (i.e., in the 1-s before receiving the ball), the frequency and excursion increased to approximately 1.5 head turns per second and 56 degrees per second, respectively.

The average frequency of players’ head movements in the current study were higher than those recorded in previous investigations (Jordet et al., 2013; Pocock et al., 2017), with the average head turn frequency in the current study being approximately 0.3 head turns/second higher than the highest average frequencies recorded by top level English Premier League players (0.62 head turns/second). This finding is likely due to the alternative definition of exploration and the use of IMU technology to quantify head movements in the current study. The definition of a head turn used in previous research would classify a look away and back to the ball as one head turn, whereas our definition would classify this as *at least* two head turns. Further, the IMU technology is able to detect smaller head movements that would not be easily detected by the human eye, which further supports a greater number of head turns being recorded with the IMU-based method. For these reasons, we would argue that the IMU-based values represent a more objective and accurate measurement of head turns than the previous method of manually counting head movements (McGuckian and Pepping, 2016; Chalkley et al., 2018).

With a shorter time-period before possession, players showed a higher HTF and HTE, indicating that, as they became closer to gaining ball possession, they explored their surroundings more extensively. This indicates exploration in support of prospective performance with the ball (Gibson, 1979; Adolph et al., 2000; Fajen, 2007; Fajen et al., 2009), as when the player recognized they were likely to receive the ball they engaged in more exploratory activity to inform their imminent actions with the ball. The changes in correlation between HTF and HTE across different time periods may indicate that these exploratory movements differed as the players came closer to receiving the ball. That is, when players were temporally further from receiving the ball (i.e., 10-s before possession) they may have used different types of exploration (e.g., using large and less frequent head movements) compared to when they were very close to receiving the ball (e.g., using small and more frequent head movements). The current analyses are unable to answer this hypothesis; however, it is an important question for future research to consider.

Across all time periods before ball possession, higher exploration excursion was related to a higher likelihood of turning with the ball or playing a pass to an area opposite to which it was received. The same was found in the final 2 s before gaining ball possession in relation to exploration frequency. Together, these findings suggest that when a player has gained more information about their environment through expansive exploratory activity, they are more likely to make use of what is afforded by their surrounding environment. This is, if a player has gained more information about the positions of surrounding teammates, opponents, and free space through their exploratory activity (i.e., they are not blind to their surroundings), they are more likely to utilize this information by turning with the ball or playing a pass behind them. Further support for this conclusion comes from the findings regarding one-touch passes. For some time-periods before ball possession, a lower HTE resulted in a higher chance of playing a one-touch pass. Here, it may be that players completed a one-touch pass *because* they had not sufficiently explored their environment, and therefore were forced to complete a one-touch pass back to a teammate because there were no other options perceived by (i.e., afforded to) them. It may also be that players were afforded with good passing options in front of them before receiving the ball, resulting in the ability to play a one-touch pass without the need to explore, however, the current analysis is unable to assess this hypothesis. Future research should include positional data to investigate the association between inter-player distances and exploratory activity.

In line with previous research, more frequent exploratory head movements resulted in a higher likelihood of a forward pass (Jordet et al., 2013); however, the strength of this association varied across different time-periods before ball possession. When players had a HTF that was higher than their individual average HTF, there were higher odds of playing an attacking pass, however, this relationship only appears when players have longer to explore (from 4 to 9 s) before gaining possession of the ball. This finding suggests that by gaining environmental information well in advance of receiving the ball, players are able to prospectively guide actions leading to an attacking pass (Adolph et al., 2000; Fajen, 2007). Interestingly, this relationship was not found when considering the HTE before receiving the ball. Although these findings are important from a performance perspective, it suggests that the relationships between exploratory actions and performance with the ball may be more complex than simple measures of head turn frequency and excursion. In fact, in such a dynamic sporting context, it is likely that the relationships between exploration and performance are also very dynamic, which may call for more sophisticated analyses in future investigations.

Head turn frequency and excursion were not associated with the successful completion of passes, regardless of the time-period before possession that was analyzed. This finding is in contrast to previous research which has reported positive associations between head turn frequency and successful pass completions (Jordet et al., 2013). In their study, Jordet et al. (2013) only included instances when the player receiving the ball was positioned between the attacking goal and the player they received the ball from. In the current study, all possessions where a pass was attempted were included. The contrasting findings may also be due to the differences in the level of expertise of participants between studies. It may be that the action capabilities of the players in the current study were a limiting factor in their ability to successfully complete more difficult passes (Fajen, 2007; Witt and Riley, 2014; Paterson et al., 2016). For example, players may have been able to perceive certain passing opportunities through extensive exploration, but their individual action capabilities (i.e., technical passing ability) may not have been reliable enough to consistently complete the afforded pass successfully. In contrast, the English Premier League players included in the study by Jordet et al. (2013) would very likely be able to reliably play a wider range of passes, resulting in a higher pass completion rate when more difficult passing opportunities were perceived.

The current study extended previous research by investigating how HTF and HTE in various time-periods before ball possession related to actions with the ball. There are, however, limitations which should be considered when evaluating the current findings. Primarily, it is important to consider that the current analysis included all offensive actions with the ball when the outcome of interest occurred. By analysing the data in this way, the specific context in which the actions with the ball occurred, such as the location on the pitch, the amount of pressure from opponents, the time of the game, the score line, the playing style of the teams involved, the individual players technical ability, etc., were not considered. It is possible that these constraints would influence the relations between exploration and performance and they should be considered in future research (Dicks et al., 2010; McGuckian et al., 2017; Oppici et al., 2017; Orth et al., 2017; Timmerman et al., 2017). For example, it may be that players are afforded different passing options in defensive areas of the pitch, which could influence the likelihood of a successful pass regardless of their exploratory actions. Further, the current sample included semi-elite players in Australia (Swann et al., 2015) and, hence, a sample of athletes with a fairly homogenous level of expertise. While similar relationships between exploration and performance have been shown in elite English Premier League players (Jordet et al., 2013), further investigations across various levels of skill and expertise are required. Using IMU technology to quantify exploratory action in future investigations will provide much needed objective data, and would effectively supplement observational data coming from video-based approaches.

Another possible focus for future research, which the current study did not investigate, is the relations between exploration and defensive actions. Thus far, research has only investigated exploratory head movement in offensive situations (Eldridge et al., 2013; Jordet et al., 2013; Pocock et al., 2017), but successful defensive performance is also influenced by one’s awareness of their surroundings. Therefore, an understanding of how exploratory head movement relates to individual actions and team structure are warranted. For example, it is likely that exploratory head movement would be related to changes in tactical exploratory movement found in small-sided games with numerical imbalances (Ric et al., 2015, 2016; Torrents et al., 2016), however, future research is needed to examine this hypothesis.

More detailed understanding of how players explore may help inform coaches about what to expect from their players during game play and before receiving the ball. Our investigation showed that the way in which players explore varies greatly between players. These differences indicate a need to individualize data collection and analysis when assessing exploratory activity in applied situations.

## Conclusion

The current investigation has demonstrated the importance of “checking one’s shoulder” before receiving the ball by investigating the head turn frequency and head turn excursion of football players in 11v11 match-play. In particular, in order for players to make successful use of their surrounding environment, the current study suggests that players must explore their environment sufficiently by employing an exploration strategy that involves high head turn frequencies and excursions. Playing a forward pass, passing to an area opposite to where the ball was received from, turning with the ball, and playing a one-touch pass were all associated with visual exploration. Hence, the advice to not “turn blind” is sound, and applied practitioners would do well to utilize these findings by designing training situations which encourage high head turn frequency and excursion in order to perform successfully. In doing so, applied practitioners may wish to implement the use of IMUs in order to easily and objectively quantify the exploratory head movements of athletes during specific training drills, thus ensuring the drills accurately represent the exploratory requirements of athletes during competitive match-play.

## Author Contributions

TM contributed to conceptualization, data collection, data analysis, and writing the paper. MC and GJ contributed to conceptualization and writing the paper. DC and G-JP contributed to conceptualization, data analysis, and writing the paper.

## Conflict of Interest Statement

The authors declare that the research was conducted in the absence of any commercial or financial relationships that could be construed as a potential conflict of interest.
